# Classification of High Curl Pattern Hair: A Systematic Review and Clinical Perspective

**DOI:** 10.1111/jocd.70828

**Published:** 2026-04-02

**Authors:** Valerie Callender, Cheryl Burgess, Valerie M. Harvey, Candrice Heath, Hope Mitchell, Heather Woolery‐Lloyd, Anneke Andriessen, Amy McMichael

**Affiliations:** ^1^ Department of Dermatology Callender Dermatology & Cosmetic Center, College of Medicine, Howard University Glenn Dale Maryland USA; ^2^ Department of Dermatology Center for Dermatology and Dermatologic Surgery, Georgetown University and the George Washington University Washington DC USA; ^3^ TPMG Hampton Roads Center for Dermatology Newport News Virginia USA; ^4^ Department of Dermatology Howard University Washington DC USA; ^5^ Mitchell Dermatology Perrysburg Ohio USA; ^6^ Skin of the Color Division University of Miami Department of Dermatology Miami Florida USA; ^7^ RBC Consultants, Anneke Andriessen & Co BV Malden the Netherlands; ^8^ Department of Dermatology Wake Forest School of Medicine Winston‐Salem North Carolina USA

## Abstract

**Background:**

Hair type classification is crucial for developing personalized hair care, maximizing hair health, and selecting appropriate treatment for scalp disorders, yet it remains underutilized in clinical practice.

**Aims:**

This review aimed to address three main questions: (1) How can hair types be defined according to specific shape criteria, without referring to race/ethnicity? (2) Why does certain hair exhibit a curly pattern, and how does curly hair behave uniquely? (3) How is the classification of curly hair clinically relevant to the development of seborrheic dermatitis?

**Methods:**

PubMed and Google Scholar were the primary and secondary platforms used for the literature search, respectively. The scope of the search included English‐language human studies published between 2000 and 2025 that investigated classification systems defining hair types based on specific shape criteria.

**Results:**

The literature review revealed four mainstream hair classification systems: the André Walker Hair Typing System, the LOIS Hair Typing System, the FIA Hair Typing System, and the L'Oréal Curl Classification System. There are shared characteristics across the systems, as each was built on previous ones to incorporate nuances of different hair types and curl patterns. The L'Oréal Curl Classification System acknowledges the broader continuum of hair types by categorizing straight, wavy, curly, and coiled hair using four morphological parameters.

**Conclusions:**

There are several existing hair classification systems that dermatologists and other health care professionals can use to optimize communication with patients. Dermatologists' use of these classification systems and increased awareness of practices for high‐curl‐pattern hair could ultimately improve treatment outcomes. Curl pattern classification may also have predictive value in the treatment of scalp diseases (e.g., seborrheic dermatitis), but further validation is required.

## 1 Introduction

Quantifying the continuous variation in human scalp hair morphology is of interest to scientific disciplines (e.g., anthropologists, geneticists, forensic scientists, and dermatologists), the beauty/hair care industry, and consumers [1]. Yet the phenotypical nature of curly human hair fibers has been rarely systematically investigated in hair research [2, 3]. Hair morphology can be examined at multiple levels, from microscopic characterization of cell structures to descriptions of the overall macroscopic appearance. The selection of variables to consider is an important distinction in describing hair form and developing hair classification systems. The selected variables must wholly and accurately describe the hair fibers [3]. Existing methods for evaluating hair fibers are time‐consuming and not widely used in everyday clinical practice (e.g., scanning electron microscopy, photogrammetry, optical microscopy). Adoption of easy‐to‐use and readily available tools could guide assessment, treatment recommendations, and improve patient compliance, yet remains underutilized in clinical practice. Therefore, this review aimed to address three main questions that are pertinent to advancing the field of curly hair science:

1. How can hair types be defined according to specific shape criteria through simple measurements, without referring to race/ethnicity?
2. Why does certain hair exhibit a curly pattern, and how does this curly hair uniquely behave?
3. How is the classification of curly hair clinically relevant, and how is it applied to the development of seborrheic dermatitis?

## 2 Methods

### 2.1 Systematic Literature Review

The literature review explored the different aspects of high‐curl patterns in human scalp hair and the “curvature fiber model,” proposed by Cloete et al. [2], as a systemized approach to investigating high‐curl pattern hair (HCPH). The “curvature fiber model” was applied during research planning to clarify the intended research focus, assist with the literature search, and highlight areas requiring further research. The “curvature fiber model” is a systemized approach to investigating curly hair [2]. The main purpose of this model is to present a systemized architecture of curly fiber elements, and it does so with three main tiers representing the formation, characterization, and behavior of curly fibers [2]. The formation level explains why certain fibers are curved, the characterization level describes the visual appearance of the fiber, and fiber behavior studies are used to determine how curly fibers deviate from their normal character under certain conditions (e.g., stress).

The primary platform used for the literature search was PubMed. Google Scholar was used as a secondary source, wherein the first 100 articles were considered. The scope of the search included human studies investigating classification systems that define hair types, such as curly hair patterns, according to specific shape criteria. The following filters were used for inclusion: English‐language publications, a publication date between January 1st, 2000, and January 30th, 2025, and full‐text availability. Eligible study types included case reports, clinical studies (phase I–IV), controlled clinical trials, equivalence trials, evaluation studies, meta‐analysis, multicenter studies, observational studies, practice guidelines, randomized controlled trials, systematic reviews, and validation studies. Excluded were publications outside the specified date range, preclinical studies, animal studies, articles not addressing the classification of hair patterns, and publications in languages other than English. The search terms used were: Human studies on classification systems that define hair types AND high curly hair patterns OR specific shape criteria OR hair types. Results were summarized in Excel (Microsoft Corporation) and reviewed by two independent reviewers. First, the titles and abstracts were reviewed, followed by a full text review. In the case of any disagreements between reviewers, a third reviewer was available for consultation.

## 3 Results

**FIGURE 1:**
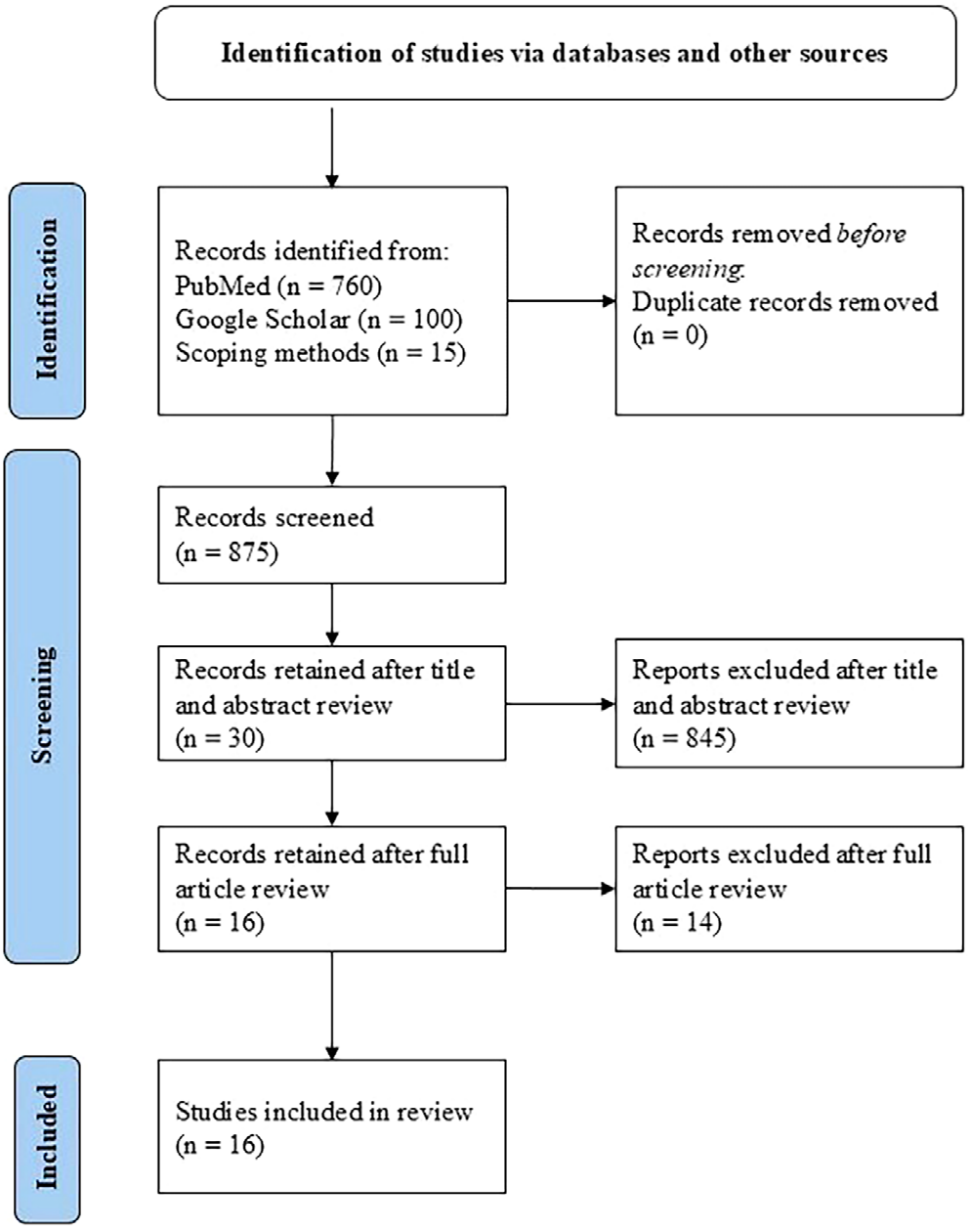
Results of the systematic literature review on the classification of hair types.

Results of the systematic literature review are presented as a PRISMA (2020) flow diagram [4], and are displayed in Figure 1. A list of the sixteen reports included is depicted in Table 1. A summary of the hair type classification systems is provided in the discussion section, and an overview of the traits considered by each system is displayed in Table 2. Based on the results of the literature review, there are four mainstream hair type classification systems:

1. The André Walker Hair Typing System
2. The LOIS Hair Typing System
3. The FIA Hair Typing System
4. The L'Oréal Curl Classification System

**TABLE 1:** Reports included in the systematic review.

No.	Citation
1	Loussouarn G, Garcel AL, Lozano I, Collaudin C, Porter C, Panhard S, Saint‐Léger D, de La Mettrie R. Worldwide diversity of hair curliness: a new method of assessment. Int J Dermatol. 2007 Oct;46 Suppl 1:2–6. doi:10.1111/j.1365‐4632.2007.03453.x. PMID: 17919196.
2	Buffoli B, Rinaldi F, Labanca M, Sorbellini E, Trink A, Guanziroli E, Rezzani R, Rodella LF. The human hair: from anatomy to physiology. Int J Dermatol. 2014 Mar;53(3):331–41. doi:10.1111/ijd.12362. Epub 2013 Dec 30. PMID: 24372228.
3	Reyes A. Curly Hair FUE: My Approach Using Classification of Follicle Curvature and Curl. International Society of Hair Restoration Surgery. 2021;31(6):205. doi:10.33589/31.6.205
4	Gaines MK, Page IY, Miller NA, et al. Reimagining Hair Science: A New Approach to Classify Curly Hair Phenotypes via New Quantitative Geometric and Structural Mechanical Parameters. Acc Chem Res. 2023;56(11):1330–1339. doi:10.1021/acs.accounts.2c00740
5	De la Mettrie R, Saint‐Léger D, Loussouarn G, Garcel A, Porter C, Langaney A. Shape variability and classification of human hair: a worldwide approach. Hum Biol. 2007 Jun;79(3):265–81. doi:10.1353/hub.2007.0045. PMID: 18078200.
6	Loussouarn G, Garcel AL, Lozano I, Collaudin C, Porter C, Panhard S, Saint‐Léger D, de La Mettrie R. Worldwide diversity of hair curliness: a new method of assessment. Int J Dermatol. 2007 Oct;46 Suppl 1:2–6. doi:10.1111/j.1365‐4632.2007.03453.x. PMID: 17919196.
7	Thibaut S, Barbarat P, Leroy F, Bernard BA. Human hair keratin network and curvature. Int J Dermatol. 2007 Oct;46 Suppl 1:7–10. doi:10.1111/j.1365‐4632.2007.03454.x. PMID: 17919197.
8	Mkentane K, Van Wyk JC, Sishi N, Gumedze F, Ngoepe M, Davids LM, Khumalo NP. Geometric classification of scalp hair for valid drug testing, 6 more reliable than 8 hair curl groups. PLoS One. 2017 Jun 1;12(6):e0172834. doi:10.1371/journal.pone.0172834. PMID: 28570555; PMCID: PMC5453415.
9	Cloete E, Khumalo NP, Ngoepe MN. The what, why and how of curly hair: a review. Proc Math Phys Eng Sci. 2019 Nov;475(2231):20190516. doi:10.1098/rspa.2019.0516. Epub 2019 Nov 20. PMID: 31824224; PMCID: PMC6894537.
10	Aslan, S., Evans, T.A., Wares, J., Norwood, K., Idelcaid, Y. and Velkov, D. (2019), Physical characterization of the hair of Mexican women. Int J Cosmet Sci, 41: 36–45. https://doi.org/10.1111/ics.12509
11	Daniels G, Fraser A, Westgate GE. How different is human hair? A critical appraisal of the reported differences in global hair fiber characteristics and properties towards defining a more relevant framework for hair type classification. Int J Cosmet Sci. 2023 Feb;45(1):50–61. doi:10.1111/ics.12819. Epub 2022 Dec 8. PMID: 36374002.
12	De la Mettrie R, Saint‐Léger D, Loussouarn G, Garcel A, Porter C, Langaney A. Shape variability and classification of human hair: a worldwide approach. Hum Biol. 2007 Jun;79(3):265–81. doi:10.1353/hub.2007.0045. PMID: 18078200.
13	Westgate GE, Ginger RS, Green MR. The biology and genetics of curly hair. Exp Dermatol. 2017 Jun;26(6):483–490. doi:10.1111/exd.13347. PMID: 28370528.
14	Nissimov JN, Das Chaudhuri AB. Hair curvature: a natural dialectic and review. Biol Rev. Camb Philos Soc. 2014 Aug;89(3):723–66. doi:10.1111/brv.12081. Epub 2014 Mar 12. PMID: 24617997.
15	Cloete E, Khumalo NP, Van Wyk JC, Ngoepe MN. Systems Approach to Human Hair Fibers: Interdependence Between Physical, Mechanical, Biochemical and Geometric Properties of Natural Healthy Hair. Front Physiol. 2019 Feb 21;10:112. doi:10.3389/fphys.2019.00112. PMID: 30846943; PMCID: PMC6393780.
16	Cloete E, Khumalo NP, Ngoepe MN. Understanding Curly Hair Mechanics: Fiber Strength. Journal of Investigative Dermatology. 2020;140(1):113–120. doi:10.1016/j.jid.2019.06.141

**TABLE 2:** Hair type classification systems and their relevant characteristics.

Hair trait	Andre Walker	LOIS	FIA	L'Oréal
**Straight**	Yes	Yes	Yes	Corresponds to Type I–II hair
1A	Yes	No	Yes	
1B	Yes	No	Yes	
1C	Yes	No	Yes	
**Wavy**	Yes	Yes	Yes	Corresponds to Type III–IV hair
2A	Yes	No	Yes	
2B	Yes	No	Yes	
2C	Yes	No	Yes	
**Curly**	Yes	Yes	Yes	Corresponds to Type V–VI hair
3A	Yes	No	Yes	
3B	Yes	No	Yes	
3C	Yes	No	Yes	
**Coils**	Yes	Yes	Yes	Corresponds to Type VII–VIII hair
4A	Yes	No	Yes	
4B	Yes	No	Yes	
4C	No	No	No	
Thickness	Not specified	Yes	Yes	No
Texture	Yes	Not specified	Yes	Yes
Volume	Not specified	No	Yes	No
Porosity	Not specified	Yes	No	No
Elasticity	No	No	No	Yes
Moisture content	No	No	No	No

### 3.1 The André Walker Hair Typing System

The “André Walker Hair Typing System” was created in the 1990's by stylist André Walker. It was originally created to market Walker's hair care products but gained popularity through celebrity endorsements. To date, it is likely the most well‐known classification system to consumers. The André Walker Hair Typing System is alphanumeric (e.g., 1B, 3C, etc.) with the numbers 1–4 denoting straight, wavy, curly, and coily textures, respectively, while the letters A, B, and C are subcategories based on curl tightness within each number (looser, medium curl, tightest curl). Although judged an improvement on some earlier systems, 3 it has been criticized for not accounting for the full range of curly hair, for overlooking that individuals can have multiple hair types on the same head, and for failing to consider other hair characteristics such as hair density or porosity [5].

### 3.2 The LOIS System

The LOIS system defines hair based on three characteristics of the hair strands: pattern, size, and texture [5, 6]. If a head of hair is dominated by right angles and bends with relatively no curve, then it is considered an “L” pattern. If strands curl or coil and appear to be shaped like the letter “O”, then it is considered an “O” pattern. If hair has no distinctive curling and lies flat against the head, then it is considered an “I” pattern. If hair strands have “S” shaped curls, then it is considered an “S” pattern [6]. The system allows combinations of the LOIS letters in case of a scalp displaying several different hair patterns.

### 3.3 The FIA Hair Typing System

The Formation, Individual Strand Thickness, Amount (FIA) Hair Typing System is similar to the Andre Walker hair typing system, as it consists of four levels of hair texture, which are differentiated by using a numbered system (1 = straight, 2 = wavy, 3 = curly, 4 = coiled). Levels 1–3 also consist of subtypes A, B, and C, and level 4 consists of subtypes A and B. FIA also introduces a consideration of hair texture into its classification system, such that hair is defined as being fine, medium, or coarse. The FIA System, unlike the Andre Walker system, takes into consideration overall hair volume, factoring in strand thickness and hair thickness (i.e., thin, medium/normal, and thick) [7].

### 3.4 The L'Oreal Curl Classification System

Unlike previous systems, which relied upon qualitative classification measures, L'Oréal developed a hair typing taxonomy based on quantitative geometric parameters displayed among the four key curl patterns (curve diameter, curl index, number of waves, number of twists). The L'Oréal Curl Classification System consists of eight types (I‐VIII), with types I–IV of curliness distinguished by curve diameter and types V–VIII further distinguished by curl index, waves, and twists. Types V–VIII correspond to curly and coily, which are regarded as high curl hair types. Thus, the use of the L'Oreal Curl Classification System promotes the inclusion of patients with curly and coiled hair by accounting for a wide spectrum of curliness. Moreover, as these geometric parameters have been correlated to the mechanical properties of hair fibers, the L'Oréal Curl Classification System acknowledges the contribution that fiber morphology has on hair fiber mechanics [8, 9].

## 4 Discussion

Traditionally, hair classification systems are consumer‐focused, and few can be translated for use in clinical practice or to guide clinical studies.

The FIA Hair Typing System is similar to the Andre Walker Hair Typing System in that it uses alphanumeric levels to define hair type, yet it goes beyond broad classification and considers specific features of the hair shaft, a characteristic shared with the LOIS System. The L'Oreal Curl Classification System considers additional variables to define hair type (e.g., curve diameter, number of waves, and curl index), and its taxonomy has been shown to correlate with the mechanical properties of hair fibers. While there are shared characteristics across all hair typing systems, each subsequent model builds on previous ones, further incorporates the nuances of different hair types, and acknowledges the broader continuum of hair qualities (Table 2).

### 4.1 The Clinical Relevance of Classification

Human scalp hair morphology (e.g., texture, form, shape, or type) is an important, but poorly understood phenotype that varies considerably within and among populations [1]. Phenotypes have often been described in ethnicity‐dependent subjective terms, which introduces bias and has traditionally been used for racial profiling and supporting racist ideologies [10]. Moreover, early classification systems fail to acknowledge that hair fibers from people of all races/ethnicities and geographies have been shown to display degrees of curl [11]. Hair density and curl pattern can even vary within an individual, depending on scalp location. For these reasons, the authors argue that current hair typing models should delineate the form of hair fibers independent of race/ethnicity [12].

The effectiveness of treatment for a variety of scalp diseases and disorders (e.g., seborrheic dermatitis, dandruff, sebum) varies across hair types and haircare practices (e.g., hair styling habits, protective treatments, chemical straightening, washing frequency, product use) [13, 14]. For this reason, hair type and lifestyle impact the suitability and ultimately, the effectiveness of treatment. A common dermatologic condition affecting hair‐bearing areas of the scalp, ears, and neck is seborrheic dermatitis (SD). SD is characterized by erythematous, scaly plaques, hypopigmentation, and hyperpigmentation in the skin of color and is often associated with pruritus. Haircare practices impact scalp health and can influence SD flares, and existing topical treatments for SD may not adequately and uniformly serve all patient populations. A recent commentary highlights key formulation concerns in topical solutions for SD [14].

The FDA has approved roflumilast topical foam, 0.3%, once daily for the treatment of SD (ages 9+). The PDE4 inhibitor is non‐steroidal, well‐tolerated, and offers an effective reduction in erythema and scaling.

In order to advance dermatological care for inflammatory diseases, such as SD, and to develop consumer products that are based on hair type and therefore more efficacious, dermatologists must forego the “one‐size fits all” approach to accommodate all hair types and haircare practices [14]. This is especially relevant for patients with highly curled/coiled hair [15], where the tightly coiled structure of the hair fibers can hinder the ability of sebum to travel down the hair shaft and moisturize the hair effectively [16]. Overall, scalp dryness, irritation, and dandruff arise from multifactorial causes, but HCPH may contribute to these concerns and also exacerbate SD symptoms. This current gap in SD management likely lowers dermatologists' confidence and accuracy in addressing hair disorders, particularly in patients with HCPH [13]. Proper classification of HCPH based on geometric parameters can help dermatologists better identify hair traits that may influence scalp diseases and disorders, potentially improving the accuracy of diagnosing and managing these disorders. Moreover, adopting a standardized hair classification system could enhance the design, inclusivity, and recruitment of clinical studies across hair types.

### 4.2 Limitations

The limitation of this review is that objective quantitative measures of classifying hair type were not considered (e.g., mechanical testing, computer vision techniques), as these methods are not practical for everyday clinician use. The limitation of many mainstream hair typing systems is that they were developed by haircare companies to promote product sales, rather than being developed or validated by independent researchers, and are not intended for use in clinical practice. It is also important to consider that, as Artificial intelligence advances, driving hair analysis technologies and platforms, hair classifications will continue to evolve, so dermatologists and other healthcare professionals must stay up to date with those innovations.

## 5 Conclusions

The authors suggest that dermatologists become familiar with existing curl pattern classification systems to optimize communication with patients and ultimately improve treatment outcomes. Curl pattern classification may have predictive value in SD patients, but further validation is required.

## Author Contributions

All authors participated in developing the supplement and reviewed the manuscript, agreeing with its content and publication.

## Funding

The authors disclose receipt of an unrestricted educational grant from CeraVe International—L'Oréal Groupe; they also received consultancy fees for their work on this project.

## Conflicts of Interest

V.C. disclosed a conflicts of interest with L'Oreal as a researcher, consultant, and speaker. The remaining authors declare no conflicts of interest with the content of this manuscript.

## Data Availability

The data that support the findings of this study are available from the corresponding author upon reasonable request.
