# Correction to “Classification of High Curl Pattern Hair: A Systematic Review and Clinical Perspective”

**DOI:** 10.1111/jocd.70869

**Published:** 2026-04-23

V. Callender, C. Burgess, V. M. Harvey, et al., “Classification of High Curl Pattern Hair: A Systematic Review and Clinical Perspective,” *Journal of Cosmetic Dermatology* 25, no. 4 (2026): e70828, https://doi.org/10.1111/jocd.70828.

When the above article was originally published, the extraneous text “Article 1:” appeared at the beginning of the title. This text has now been removed from the title.

We apologize for this error.

